# Cross-Talk between Wnt and Hh Signaling Pathways in the Pathology of Basal Cell Carcinoma

**DOI:** 10.3390/ijerph15071442

**Published:** 2018-07-09

**Authors:** Felicite K. Noubissi, Clement G. Yedjou, Vladimir S. Spiegelman, Paul B. Tchounwou

**Affiliations:** 1 Department of Biology, Jackson State University, 1400 Lynch Street, Box 18540, Jackson, MS 39217, USA; clement.yedjou@jsums.edu (C.G.Y.); paul.b.tchounwou@jsums.edu (P.B.T.); 2 Research Centers in Minority Institutions Center for Environmental Health (RCMI-CEH), Jackson State University, 1400 Lynch Street, Box 18540, Jackson, MS 39217, USA; 3 Natural Chemotherapeutics Research Laboratory, Jackson State University, 1400 Lynch Street, Box 18540, Jackson, MS 39217, USA; 4 Division of Pediatric Hematology/Oncology, Department of Pediatrics, P.O. Box 850, M.C. H085, Pennsylvania State University, Hershey Medical Center, Hershey, PA 17033, USA; vspiegelman@pennstatehealth.psu.edu

**Keywords:** basal cell carcinoma, Hh, Wnt, cross-talk, IGF2BP1, GLI1, therapeutic mechanisms

## Abstract

Basal cell carcinoma (BCC) is the most frequently occurring form of all cancers. The cost of care for BCC is one of the highest for all cancers in the Medicare population in the United States. Activation of Hedgehog (Hh) signaling pathway appears to be a key driver of BCC development. Studies involving mouse models have provided evidence that activation of the glioma-associated oncogene (GLI) family of transcription factors is a key step in the initiation of the tumorigenic program leading to BCC. Activation of the Wnt pathway is also observed in BCCs. In addition, the Wnt signaling pathway has been shown to be required in Hh pathway-driven development of BCC in a mouse model. Cross-talks between Wnt and Hh pathways have been observed at different levels, yet the mechanisms of these cross-talks are not fully understood. In this review, we examine the mechanism of cross-talk between Wnt and Hh signaling in BCC development and its potential relevance for treatment. Recent studies have identified insulin-like growth factor 2 mRNA-binding protein 1 (IGF2BP1), a direct target of the Wnt/β-catenin signaling, as the factor that binds to GLI1 mRNA and upregulates its levels and activities. This mode of regulation of GLI1 appears important in BCC tumorigenesis and could be explored in the treatment of BCCs.

## 1. Introduction

Basal cell carcinoma (BCC) is the most common form of cancer, affecting more than two million Americans each year. The vast majority of BCCs occur sporadically, but patients with the rare heritable disorder, basal cell nevus syndrome (BCNS), have marked susceptibility to developing BCCs [1]. BCC used to be more common in people over age 40, but is now also diagnosed in the younger population [2,3,4]. BCC lesions occur mostly on areas of skin that are regularly exposed to sunlight or other ultraviolet radiation, particularly the face, ears, neck, scalp, shoulders, and back. Meanwhile, BCC has also been reported from virtually every part of the skin surface [5]. Anyone with a history of sun exposure can develop BCC. However, people who are at highest risk are the ones in the fair skinned populations [6,7,8]. Immunocompromised patients have also been reported to have a higher risk of developing BCC than the general population [9,10] and BCCs appear to show a more aggressive behavior in these patients [11]. Chronic exposure to arsenic might also be responsible for the incidence of BCC development [12,13,14]. BCCs can be generally classified in four groups according to the form of growth pattern. These groups are: (i) the nodular including the micronodular form; (ii) the infiltrative including the morphoeic form; (iii) the superficial, apparently multicentric form, and (iv) the mixed form including a combination of any two or all of the types. Micronodular, infiltrative; and morpheaform patterns often show a greater aggressive behavior [2,3]. Primary lesions in the head and neck have been shown to recur frequently, while those affecting the ears, the genital organs, and other mucosal surfaces show a high propensity to metastasize (reviewed in [15]). Although the death rate from BCCs is low as BCC rarely metastasizes [5,16], if untreated BCC can destroy the tissues and nearby organs, causing ulceration and disfigurement. Advanced BCCs are resistant to treatment and associated with high morbidity [1,17,18,19]. BCC morbidity is considerable and constitutes a huge burden on the healthcare service worldwide. The cost of care for BCC patients is one of the highest for all cancers in the Medicare population in the United States [15]. In addition, people who develop BCC are predisposed to developing further BCCs and other malignancies [20].

The Hh signaling pathway has been identified as one of the drivers of BCC development [1,21,22]. Drugs targeting this pathway have been developed in the treatment of advanced, inoperable, or metastatic BCCs. Those drugs have been particularly directed against smoothened (SMO), which is an important component of the Hh pathway. Although this treatment method has been proven beneficial in many cases [23,24], the development of resistance remains a major concern as about 20% of patients develop resistance during the first year of treatment [19,23,25]. Studies have now shown that other pathways interact with the Hh pathway in BCC development [26,27,28,29,30,31,32]. Interestingly, the Wnt/β-catenin signaling pathway is one of those pathways. Investigating those interactions could be valuable in identifying better strategies in the treatment of advanced, inoperable and/or resistant BCCs. In this review, we have discussed specifically what is known on the cross-talk between Hh and Wnt signaling pathways in BCC development, based on the literature covering the last two decades and the potential relevance of this cross-talk in the treatment of SMO inhibitors’ resistant BCCs.

## 2. BCC and Hh Signaling Pathway

**Figure 1:**
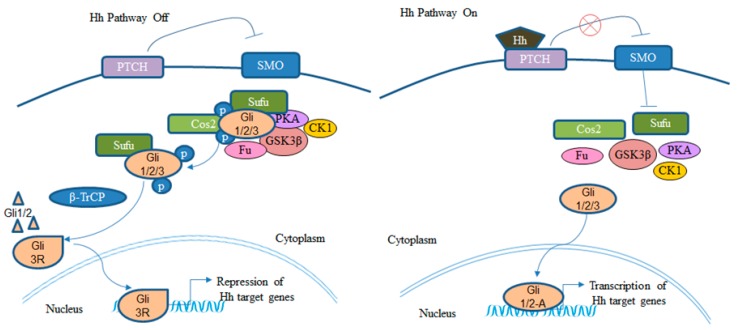
The canonical Hh signaling pathway. GLI 3R; GLI 3 repressor; GLI 1/2-A; GLI 1 and 2 activators [34,42].

**Figure 2:**
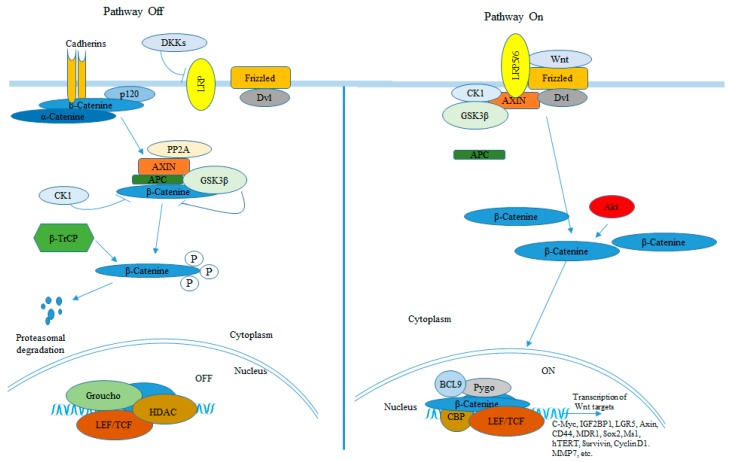
The canonical Wnt/β-catenin signaling pathway [55,56,57,58,60,64,66,68].

**Figure 3:**
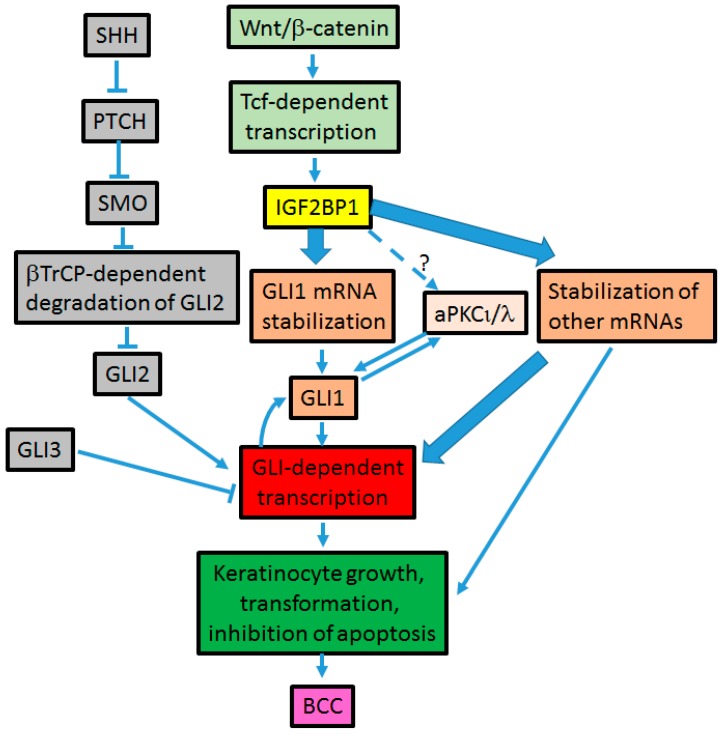
Cross-talk between Wnt and Hh signaling pathways [27,41,88,89,90]; 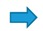 mechanisms of insulin-like growth factor 2 mRNA-binding protein (IGF2BP1) -driven BCC tumorigenesis; 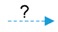 potential mechanism.

The Hh signaling is a critical pathway governing embryonic development and stem cell maintenance. However, its aberrant regulation is implicated in the development of many cancers including BCC [33]. The Hh pathway is mediated by the Ci/GLI family of zinc finger transcription factors. The transmembrane receptors Patched (PTCH) and Smoothened (SMO) play critical roles in the Hh signaling. In the absence of the Hh ligand, its receptor PTCH inhibits the function of SMO, thereby inactivating the Hh signaling. When the Hh ligand binds to PTCH, the inhibitory effect of PTCH on SMO is removed leading to the activation of the transcription factor Ci/GLI (Figure 1) (reviewed in [34]). Three GLI genes have been identified in vertebrates. GLI1 being predominantly a transcriptional activator, whereas GLI2 and GLI3 can perform as both activators and repressors [35,36]. Activating mutations of SMO or suppressing mutations of PTCH have been shown to constitutively activate the Hh signaling pathway [33]. Studies have also shown that constitutive activation of Hh signaling pathway is a key factor driving the development of BCC [1,37]. Loss of function mutation of the *PTCH* gene and gain of function mutation of the *SMO* gene have been observed in BCCs [21,22]. Indeed, patients with the autosomal dominant nevoid basal cell carcinoma syndrome are predisposed to BCC and medulloblastoma since they have inherited mutations in one allele of the *PTCH1* gene, and BCCs from these patients lack the normal *PTCH1* gene [38]. Over-expression of Sonic Hedgehog (SHH) has been shown to result in the formation of BCC in murine studies [37], and activation of GLI1 has been identified as a key step in the initiation of the tumorigenic program leading to BCC [39]. GLI1 is highly expressed in human BCC [40]. Therefore, inhibiting GLI1 expression and activity would be a critical step towards BCC treatment. Over-expression of GLI1 in BCCs appears modulated not only by the upstream Hh signaling but also by alternative mechanisms [30,31] (Figure 2 and Figure 3). This warrants the exploration of those mechanisms, especially their interaction with the Hh pathway while developing new strategies in BCC treatment. One such mechanism modulating GLI1 expression and activity is Wnt/β-catenin-dependent [27,41].

## 3. BCC and Wnt/β-Catenin Signaling Pathway

The Wnt signaling is another pathway implicated in basal cell carcinoma development. Similarly to the Hh pathway, the Wnt signaling pathway plays a critical role in patterning and cell proliferation of embryonic and adult tissues. However, aberrant activation of the Wnt signaling pathway is responsible for the development of many human cancers as well [43]. β-catenin is a pivotal player in the signaling pathway initiated by Wnt proteins. In the absence of Wnt signaling, β-catenin, which is found in a complex together with axin, adenomatous polyposis coli (APC), and glycogen synthase kinase β (GSK3β), is phosphorylated by GSK3β and subsequently ubiquitinated and degraded in the proteasomes. Binding of Wnt proteins to the receptors Frizzled and LDL-receptor related protein (LRP) families on the cell surface leads to GSK3β inactivation resulting in the release of unphosphorylated β-catenin from the multiprotein complex. β-catenin is then translocated into the nucleus, where it binds to Tcf/Lef causing the activation of Wnt target genes (Figure 2). Loss of function mutation of the tumor suppressor APC, stabilizing mutations of β-catenin, or mutations in axin result in constitutive activation of the Wnt signaling pathway [44]. Aberrant activation of the canonical Wnt/β-catenin signaling pathway has been implicated in the development of many cancers including colorectal cancer (reviewed in [45]), breast cancer (reviewed in [46]), and melanoma [47,48]. Activation of the Wnt pathway has also been observed in BCCs as shown by over-expression of Wnt proteins [49,50] and the presence of β-catenin harboring stabilizing mutations [51]. Additionally, cytoplasmic and/or nuclear localization of β-catenin have been observed in different human BCC tumors [50,52,53,54].

Wnt/β-catenin signaling is known to promote cell growth, morphogenesis, and stem cell maintenance. It is also one of the major pathways stimulating the stemness of a rare population of cells in the tumor bulk responsible for the resistance of cancer to conventional chemo treatment, resulting in the relapse of the disease and metastasis. The Wnt/β-catenin sustains the proliferation and self-renewal capabilities of those particular cells through regulation of its many targets. hTERT is one of the Wnt/β-catenin signaling direct targets. By upregulating hTERT expression and activity in cancer cells, the Wnt/β-catenin signaling stimulates their immortality [55,56], their invasion and metastasis capabilities [57]. Aberrant activation of the Wnt/β-catenin signaling was also shown to upregulate BIRC5/Survivin [58,59], which in turn inhibits caspase-3 and -7 and prevents apoptosis adding to the immortality of cancer cells. Musashi-1 [60,61,62,63], and Sox2 [64,65] are markers of stem cells that are overexpressed in many cancers and contribute to their tumorigenic phenotype by stimulating cell proliferation and drug resistance, respectively. These stem cell markers have been demonstrated to be upregulated by the Wnt/β-catenin signaling. The Wnt/β-catenin signaling also promotes drug resistance by regulating other genes including MDR-1 [66,67], CD44, MMP7 [68] and IL-10 [69]. The Wnt/β-catenin regulates many genes involved in cell cycle progression, migration, invasion, and extracellular matrix remodeling. However, these Wnt targets have not been specifically identified in BCC. But, they could possibly contribute to the resistance of advanced BCC to treatment. Studies have shown that inhibition of the Wnt/β-catenin signaling sensitizes cancer cells to chemotherapeutic drugs [70,71,72] and could improve the efficacy of immunotherapy [73,74]. Therefore, targeting the Wnt pathway could be an effective approach in the treatment of advanced, inoperable and/or resistant BCCs.

## 4. Cross-Talk between Hh and Wnt Signaling Pathways in BCC Development (Figure 3)

Interaction between Hh and Wnt signaling has been shown to be fundamental in the coordination of key processes during embryonic development, stem cell maintenance, and tumorigenesis. However, the mechanisms of those interactions are still not fully understood. In normal skin, both Wnt and Hh signaling are required during hair follicle morphogenesis as Wnt signaling initiates hair bud formation, whereas Hh signaling proliferates the follicle epithelium needed to form a mature follicle [75,76,77,78,79]. Cross-talk between Wnt and Hh signaling seems to exist in skin pathogenesis as well. Upregulation of Wnt genes by GLI1 was shown in BCC-like tumors in late tailbud tadpole stages [49]. Additionally, Wnt proteins were found to be overexpressed in human BCC tumors [49,50]. The first evidence of a requirement of the canonical Wnt signaling pathway in Hh pathway-driven development of BCCs was shown in a BCC mouse model, *M2SMO* [80]. The study demonstrated that blockade of the canonical Wnt signaling prevented Hh signaling-driven tumorigenesis [80]. Wnt and Hh are two major pathways that have been postulated to interact at multiple levels [49,81,82,83,84,85,86,87,88], yet the mechanisms of these interactions are not clear. Understanding the molecular mechanisms by which both pathways relate to each other in BCC would be critical in the perspective of developing novel therapeutic approaches in the treatment of this disease and alleviate the cost of care for BCCs.

In our previous studies, we demonstrated that the mechanism of cross-talk between Hh and Wnt signaling pathways in BCC development employs GLI1 mRNA stabilization by the insulin-like growth factor 2 mRNA-binding protein (IGF2BP1) [41]. IGF2BP1 (also known as IMP1, CRD-BP, and ZBP1) is a direct target of the canonical Wnt/β-catenin signaling pathway [89]. We showed that IGF2BP1 binds to a segment of the coding region of GLI1 mRNA and stabilizes it. We also demonstrated that Wnt/β-catenin signaling induces the expression and transcriptional activity of GLI1 in an IGF2BP1 dependent manner [41]. The binding region of IGF2BP1 on GLI 1 mRNA that we identified was narrowed to a 61-nucleotide segment in a different study and was shown to exhibit two characteristic stem-loop motifs [90]. The use of GLI1 mRNA competitors harboring the two stem-loops effectively prevented GLI1 mRNA-IGF2BP1 interaction in vitro. Knockdown of IGF2BP1 in colorectal and breast cancer cell lines using the RNAi approach significantly lowered GLI1 expression in these cells. Furthermore, the RNA oligo that effectively competed with GLI1 mRNA-IGF2BP1 interaction was efficient at reducing GLI1 expression in colorectal and breast cancer cells [90]. IGF2BP1 is over-expressed in BCC and its expression positively correlates with the activation of both Wnt and Hh signaling pathways [27]. 

One of the obstacles in studying BCC development is the absence of human BCC cell lines. We developed and characterized a human BCC cell line, the UW-BCC1 cell line. IGF2BP1 is highly expressed in this cell line. Knockdown of IGF2BP1 in this cell line using a specific IGF2BP1 shRNA significantly reduced its growth, proliferation, invasion capability, as well as promoted its apoptosis. Inhibition of IGF2BP1 had a more pronounced effect on the tumorigenic phenotype of this cell line than inhibition of GLI1, probably due to the pleiotropic effect of IGF2BP1 on other oncogenes including c-myc and β-TrCP1. Collectively, the regulation of GLI1 expression and activities by IGF2BP1 plays an important role in BCC development [27]. A significant clinical relevance of the mode of action of IGF2BP1 for BCC treatment is that the regulation of GLI-dependent transcriptional activity by IGF2BP1 seems SMO independent [41]. Additionally, inhibition of IGF2BP1 appears to prevent *PTCH* mutant driven GLI transcriptional activity [27]. This implies that the Hh signaling could be inhibited by downregulating IGF2BP1 and therefore bypassing SMO. Additional studies using BCC cell lines are warranted to further confirm this novel mechanism that could be explored in the development of new treatment strategies for BCCs especially for SMO inhibitor resistant BCCs.

A previous study showed that IGF2BP1 binds to the coding region of β-TrCP1 mRNA and shields it from degradation by a microRNA [91]. A large number of microRNAs has been identified in BCCs, some of which are tumor suppressors [92]. IGF2BP1 might achieve its oncogenic property in BCCs by binding to GLI1 mRNA and shielding it from degradation by a microRNA as well. This possibility should be explored for BCC treatment.

## 5. BCC Treatment

Surgical resection and/or radiation therapy is the mainstream of treatment for small, early stage, and localized BCCs. However, different approaches are used in the treatment of BCC depending on the BCC subtype, its stage of development, and its location. Imiquimod [93,94,95,96] and topical 5-fluorouracil [97] have been approved in the treatment of superficial BCCs. The photodynamic therapy [98,99] is another approach used in the treatment of nodular and superficial BCCs. However, treatment options for advanced and metastatic BCCs are still emerging. New drugs approved or in clinical trials for the treatment of advanced or metastatic BCCs include the SMO inhibitors sonidegib [100,101] and antifungal itraconazole [102]. The systematic use of vismodegib has proven successful in many cases [23,24,103,104]. Vismodegib is the SMO inhibitor that is approved by the Food and Drug Administration (FDA) for the treatment of advanced/inoperable and metastatic BCCs [23]. Although the use of this drug showed a significant response rate (48% in advanced and metastatic BCCs) [23,24], about 20% of patients presenting advanced or metastatic BCC develop resistance during the first year [105]. Drug resistance is an obstacle that impairs the success of cancer therapies. Recent studies demonstrated that vismodegib resistant BCCs are still addicted to the Hh pathway and that mutation in the ligand binding pocket of SMO is one of the mechanisms developed by BCC to resist treatment to vismodegib [31,106]. The use of some GLI antagonists downstream of SMO was found to be effective in suppressing the Hh pathway in SMO inhibitor resistant BCCs. Those antagonists include an inhibitor of atypical Protein Kinase C-iota/lambda (aPKC-ι/λ) that phosphorylates GLI1, and arsenic trioxide that inhibits GLI2 [31]. Therefore, targeting factors downstream of SMO was proposed as a better approach to overcome resistance [31]. IGF2BP1 regulates GLI1 downstream of SMO and appears to be an attractive candidate gene to target in the treatment of BCCs including SMO inhibitor resistant BCCs.

IGF2BP1 is a multifunctional RNA-binding protein involved in different processes such as mRNA turnover, translation control, and localization. IGF2BP1 expression is temporally and spatially regulated. IGF2BP1 is abundantly expressed in fetal and neonatal tissues. Its expression is crucial in the regulation of many transcripts essential for normal embryonic development [107,108,109]. While scarce or absent in normal adult tissues, IGF2BP1 is found to be de novo activated and/or overexpressed in various cancers [48,89,108,109,110,111,112,113]. Its expression was shown to be associated with the most aggressive form of some cancers [108,111,114]. IGF2BP1 also regulates other factors implicated in cancer development, metastasis, and resistance to drug including c-myc [115], MDR1 [116], β-TrCP1 [89], CD-44 [117], and cIAP1 [118]. We showed that Hh signaling could be inhibited by downregulating IGF2BP1 and this mechanism bypasses SMO [41]. This mode of regulation of oncogenes by IGF2BP1 portrays it as an attractive target in the treatment of BCCs including SMO inhibitor resistant BCCs. IGF2BP1 binds to its targets to regulate their expression and activities. Identifying small molecules that prevent IGF2BP1-GLI1 mRNA interaction would be beneficial in BCC treatment.

## 6. Conclusions

Although the number of new cases of BCC is still on the rise each year, the molecular mechanisms driving its development are still unclear. A cross-talk between Wnt and Hh signaling pathways appears to play a significant role in BCC development. This cross-talk is modulated by IGF2BP1, which activates GLI1, the key element driving BCC development. Inhibiting IGF2BP1 or preventing its interaction with GLI1 mRNA seems to represent a sound approach in BCC treatment. However, further studies including in vivo studies to delineate the contribution of IGF2BP1 to BCC development will bring new insights on the potential candidacy of IGF2BP1 as a target for therapy. This may potentially lead to the designing of new agents effective in the treatment of BCCs, including advanced, inoperable, or SMO inhibitor resistant BCCs. This would alleviate the cost of care for BCCs and reduce the morbidity associated with the advanced form of this disease. Additionally, this would represent a better alternative to multiple surgical procedures especially for immunocompromised patients and patients with basal cell nevus syndrome who can develop tens to hundreds of BCCs in their lifetime.
